# Unilateral Auditory Cortex Lesions Impair or Improve Discrimination Learning of Amplitude Modulated Sounds, Depending on Lesion Side

**DOI:** 10.1371/journal.pone.0087159

**Published:** 2014-01-23

**Authors:** Holger Schulze, Anke Deutscher, Konstantin Tziridis, Henning Scheich

**Affiliations:** 1 Experimental Otolaryngology, University of Erlangen-Nuremberg, Erlangen, Germany; 2 Leibniz Institute for Neurobiology, Magdeburg, Germany; Nathan Kline Institute and New York University School of Medicine, United States of America

## Abstract

A fundamental principle of brain organization is bilateral symmetry of structures and functions. For spatial sensory and motor information processing, this organization is generally plausible subserving orientation and coordination of a bilaterally symmetric body. However, breaking of the symmetry principle is often seen for functions that depend on convergent information processing and lateralized output control, e.g. left hemispheric dominance for the linguistic speech system. Conversely, a subtle splitting of functions into hemispheres may occur if peripheral information from symmetric sense organs is partly redundant, e.g. auditory pattern recognition, and therefore allows central conceptualizations of complex stimuli from different feature viewpoints, as demonstrated e.g. for hemispheric analysis of frequency modulations in auditory cortex (AC) of mammals including humans. Here we demonstrate that discrimination learning of rapidly but not of slowly amplitude modulated tones is non-uniformly distributed across both hemispheres: While unilateral ablation of left AC in gerbils leads to impairment of normal discrimination learning of rapid amplitude modulations, right side ablations lead to improvement over normal learning. These results point to a rivalry interaction between both ACs in the intact brain where the right side competes with and weakens learning capability maximally attainable by the dominant left side alone.

## Introduction

A fundamental principle of brain organization is a mosaic type division of cortex into numerous functional areas which cooperate in various combinations during cortex-dependent behaviour. Functionally defined areas like auditory cortex (AC) are further subdivided into a number of fields or sub-areas within these fields. They reach their functional specialization partially by their connections with different subcortical structures and nuclei and partially by cortico-cortical connections [1], [2]. Functions are also divided between left and right AC. The full extent of this division of labour between the hemispheres may not become apparent by selective representation of stimulus features upon parametric stimulus exposure but by behaviourally solving different auditory tasks [3], [4], [5], [6].

In a preceding study of bilateral lesions of gerbil AC we have demonstrated that discrimination learning even within a physically continuous class of auditory stimuli, i.e. amplitude modulations (AM), may involve different brain and AC regions when this stimulus class encompasses discontinuous classes of percepts [7]. When the repetition rate of AM tones gradually accelerates, the initially perceived rhythmic intensity changes fuse into an intermediate range of hoarse sounds (roughness percept), which then change to tonal sounds with rising pitch corresponding to the repetition rate. The repetition rate (periodicity) of such fast AM stimuli is represented in gerbil primary AC field AI by a special type of topographic map of neurons characterized by a cyclic functional gradient of best modulation frequency. For slow AMs, by contrast, there is no specialized cortical map and their processing is widely distributed in AI [8], [9], [10]. The study by Deutscher et al. [7] showed that after bilateral AC lesions the go/no go shuttle box discrimination in the range of slow AMs could still be performed by subcortical processing whereas discrimination of fast AM required intact cortical processing. Note also that discrimination learning of slow AM is significantly faster than discrimination learning of fast AM [11] (cf. Results).

Here we demonstrate with unilateral AC lesions that this cortical discrimination learning of fast AM is not uniformly distributed across the two hemispheres, but predominantly involves the left AC. Furthermore, while unilateral ablation of the left AC leads to an impairment of normal AM discrimination learning, we show that right AC ablation results in an improvement over normal learning. This result points to an interaction between the ACs of the two hemispheres in the intact brain where the right hemisphere competes with and weakens AM discrimination learning maximally attainable by the dominant left hemisphere alone.

## Materials and Methods

### Ethics Statement and Animals

Experiments that fulfilled all procedural and lesion size criteria covered 83 adult male Mongolian gerbils from our colony (age at the beginning of the experiments 3 to 6 month). All experiments were conducted in accordance with the NIH Guidelines for Animals in Research and with the ethical principles defined by the German law for the protection of experimental animals. The experimental protocols were approved by an ethics committee of the state of Sachsen-Anhalt, Germany.

### Behavioural Training, Training Groups

A foot shock motivated shuttle-box go/no go avoidance procedure (UCS; unconditioned stimulus; 150 to 300 µA) was used for discrimination training of two sinusoidal AM tones with 100% modulation depth and different periodicities but identical carrier frequency of 2 kHz. The auditory stimulation was provided via two speakers in the ceiling of the shuttle-box about 20 cm above the floor grid. All sounds were presented with 60 dB SPL measured in 15 cm distance from the speaker (note that due to reflections within the shuttle-box the sound intensity may vary with location). Stimuli had 400 ms duration with 5 ms rise and fall time and were presented repeatedly at 2 Hz. Naïve gerbils were trained in daily sessions over 15 days to discriminate periodicities of either 20 Hz vs. 40 Hz or 160 Hz vs. 320 Hz [7], [11]. The lower periodicity always served as the conditioned stimulus (CS+; conditioned stimulus followed by the foot shock UCS) that required jumping the hurdle (height: 4 cm), whereas the higher periodicity served as the conditioned stimulus (CS–; conditioned stimulus not followed by the UCS) that required abstaining from jumping the hurdle. Within each session, 30 trials of each stimulus (CS+ or CS–) were presented in pseudo-randomized order. Variable intertrial intervals (start to start) were 16+/−4 sec. Crossings of the hurdle during a 4 sec presentation of the CS+, i.e. correct conditioned responses (CR+) and false alarms during the 4 sec presentation of the CS– (CR-), respectively, were counted in each session. If the animal did not cross the hurdle within 4 sec after the onset of the CS+, the UCS was turned on and the CS+ presentation continued until the gerbil crossed the hurdle, but maximally for 8 sec. An additional UCS (duration 0.5 sec) was applied on the other side of the hurdle upon false alarm to the CS–. Strength of the UCS ranged between 100 and 450 µA and was adapted individually to each animal to be unpleasant (search for escape) but not painful to avoid fear behaviour (freezing). Polarity of the steel bars of the floor grid was varied randomly to avoid learning of the polarity pattern of the floor grid by the animals.

Animals were divided in sub-groups that received cortical AC ablation prior to the first training session on the right or left side or sham surgeries, respectively. Sham-lesioned animals with complete surgery but without cortical ablation served as controls. Because there were no significant differences in the learning performance of left or right sham-lesioned animals, these were combined into one single control groups for each training paradigm, consisting of equal amounts of left or right sham-lesioned animals, respectively. This resulted in a total of 6 experimental groups: left lesion, right lesion and control group, trained to either 20 vs. 40 Hz or 160 vs. 320 Hz periodicity, respectively. Training was carried out as described in detail earlier [11].

### Cortical Ablation

Methods were similar to our earlier studies with auditory cortex lesions [6], [12], [13]. Surgery was performed under deep general anaesthesia by an intraperitoneal infusion of ketamine (50 mg/ml; Ratiopharm), xylazine (Rompun 2%, BayerVital) and isotonic sodium chloride solution (mixture 9∶1∶10) at a rate of 0.06 ml/h, after an initial dose of 0.2 ml. Body temperature was maintained at 37°C using a remote-controlled heating blanket. The skin over the temporal bone on the ablation side was cut and retracted; the musculature covering the temporal bones was partly removed. The AC was then exposed by craniotomy and thermocoagulation was performed using a fine tip soldering iron centred to the AI. Using described landmarks the lesion attempted to cover AI and the surrounding fields of gerbil AC which comprise an area of approximately 8 to 9 mm^2^ [14], [15]. Finally, the skin over the trepanation area was sealed again using Histoacryl (Braun). After surgery, animals showed no sign of pain or distress or any altered behaviour compared to pre-surgery behaviour. They were given a recovery time of two days before behavioural training started.

### Histology

**Figure 1:**
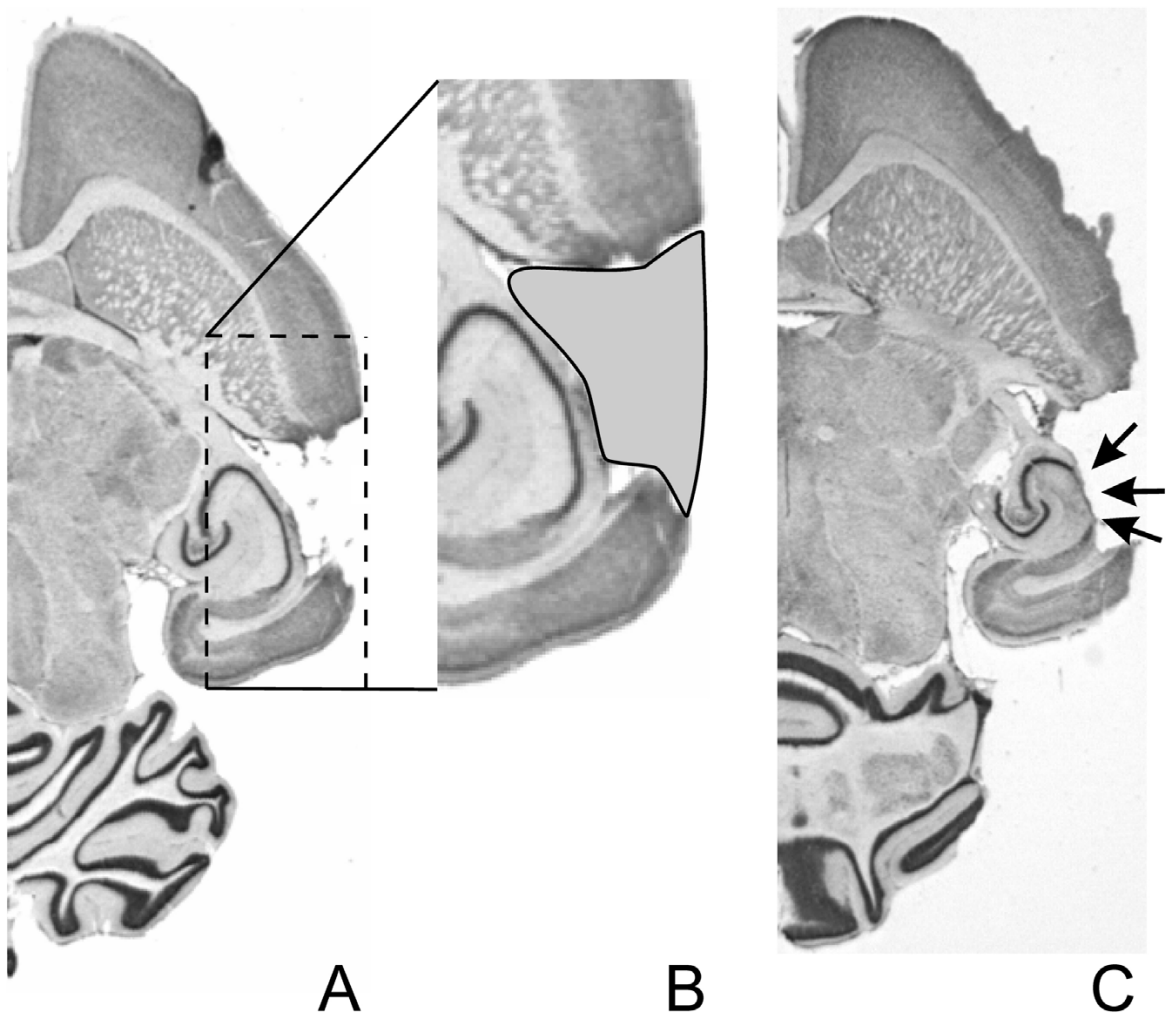
Histological verification of lesion size and location. Shown are two examples (A and C) of Nissl-stained horizontal brain sections. **A** For animals in which no damage to the hippocampus could be detected. **B** The contour of the lesion was determined manually for later computation of lesion volume (cf. Methods). **C** Animals that showed damage to the hippocampus (the arrows point to damage of parts of the alveus, stratum oriens and stratum pyramidale of the hippocampus) were excluded from further analysis.

For histological verification of lesion size, Nissl-stained brain sections (Figure 1) were obtained from each cortex-ablated animal. After the last training session, animals were killed by an intrapulmonary injection of T61 (Intervet) and then decapitated. Brains were removed, mounted on object slides with 8% gelatine and frozen (−50°C isopentane). Horizontal sections of 40 µm were obtained using a cryostat (−6°C object temperature; −15°C blade temperature) and stained with cresyl violet [16]. Only animals with unilaterally ablated AC, but without damage of surrounding tissue like the hippocampus or striatum, were included in further analysis of behavioural data.

### Estimation of Lesion Volume

Lesion volumes were estimated from Nissl-stained brain sections [16]: Series of horizontal sections of each brain containing the complete lesion were camera-scanned (Leica M420, Grundig FA87d, Germany) and mounted using a two-dimensional computer-aided graphic program (N.I.H.-Image program by W. Rasbande, Adobe Photoshop, Macintosh). The contour of the lesion was determined manually (cf. Fig. 1B), the lesioned outer cortical border was interpolated from the remaining cortical surface. Lesion volume was computed by multiplying the total lesion area as calculated by the software with the slice thickness of 40 µm. Depending on lesion size, a lesion typically encompassed 60 to 80 Nissl-sections. The lesion size of all lesioned animals was finally analysed by a generalized k-means cluster analysis and divided into small and large lesions with the threshold of 7 mm^3^ that resulted from the cluster analysis.

### Statistical Analysis of Behavioural Data

To quantify the behavioural learning data we used measures of how fast and how well gerbils learned to discriminate between CS+ and CS–: As an index for learning speed (LS) we determined for each individual animal, the first session in which the responses to the CR+ were significantly different from those to the CR- on the 1% level (fourfold table χ2-test). Responses were hit (hurdle crossing in response to CS+), miss (no response to CS+), false alarm (hurdle crossing in response to CS -) and correct rejection (no response to CS–). This session had to be followed by a session that also fulfilled this criterion. We further analysed all data statistically with nonparametric tools as Kolmogorov-Smirnov tests rejected normal distributions of the learning parameters in all animal groups with p<0.05. Generally, all parameters tested between two groups were assessed by two-tailed Mann-Whitney U-tests, between several groups with Kruskal-Wallis ANOVA and within one group with Wilcoxon tests. The discrimination performance (DP) was calculated on a daily basis subtracting CS– from CS+. We determined a measure of the final discrimination performance (final DP): The median of the difference between CR+ and CR- that was reached during the last 8 training sessions by each individual gerbil. We chose the last 8 sessions because post-hoc tests in Kruskal-Wallis ANOVAs showed no further changes in CR+ or DP in all 6 groups. Between groups, these measures were compared using the Mann-Whitney U-test; CR- was analysed in a similar manner. Furthermore nonlinear regression analyses were used to compare the learning dynamics (CR+ -functions) and the DP of the different groups over all sessions: We compared the regression fits of left and right lesion groups with the appropriate control group over time. Additionally, Mann-Whitney-U tests were used for comparing CR+ and DP values across the first 7 days of training to investigate minor differences between groups that cannot be assessed by the nonlinear regression analyses. The p-values were corrected for multiple comparisons (Bonferroni) where needed.

## Results

Results presented in this report are based on behavioural learning data from a total of 83 animals with lesions within tolerable limits. A typical unilateral example of such a lesion that only comprises cortical tissue is shown in Figure 1A and B. A lesion as shown in Figure 1C that expanded into the hippocampus posed an exclusion criterion and data from animals with such lesions were discarded from further analysis. Out of the total number of animals, 38 were trained to discriminate AM tones with periodicities of 20 vs. 40 Hz (left AC lesion: N = 11; right AC lesion: N = 9; control: N = 18), and 45 were trained to discriminate AM tones with periodicities of 160 vs. 320 Hz (left AC lesion: N = 13; right AC lesion: N = 12; control: N = 20).

**Figure 2:**
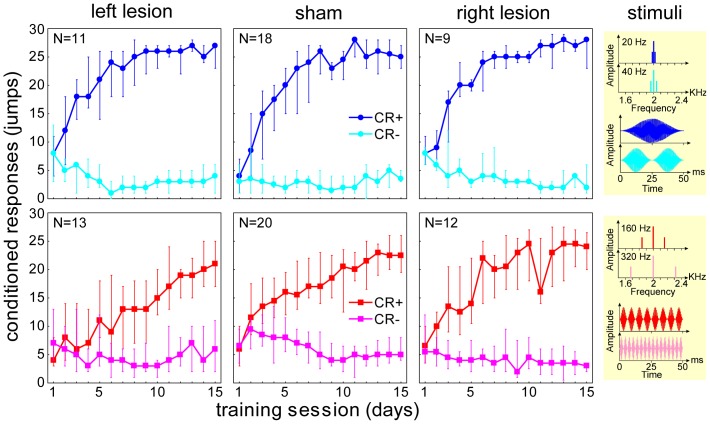
Discrimination learning curves of left and right AC lesion and control groups. Large panels show medians and quartiles of conditioned response counts as a function of daily training sessions. Each session consisted of 60 trials with 30 pseudo-randomized presentations of each stimulus (30 CR+ equal 100% correct responses, 30 CR- equal 100% false alarms). Panels in top row (blue data points) give learning curves of animals that were trained to discriminate periodicities of 20 Hz ( =  CS+) vs. 40 Hz (CS–), panels in bottom row (red data points) those of animals that were trained to discriminate periodicities of 160 Hz ( =  CS+) vs. 320 Hz (CS–). Yellow panels on the right side show spectrum and waveform of the training stimuli.

Comparing the learning data from the groups that received unilateral AC lesion with the respective control groups (Figure 2), no differences in learning performance are obvious for the animals that were trained to discriminate slow periodicities of 20 vs. 40 Hz (top panels, blue curves): Nonlinear regression analysis between CR+-curves of left or right lesioned groups vs. the control group showed no significant differences in LS across the first 7 training days (Left vs. control: Beta = 0.02, t(414) = 0.66, p = 0.50; Right vs. control: Beta = 0.01, t(383) = 0.31, p = 0.75). In addition, Mann-Whitney U-tests across the first seven days detected no significant differences (given is the median (lower quartile, upper quartile): Left (18 (11, 24)) vs. control (16 (9, 22)), p = 0.13; Right (19(11, 23)) vs. control (16 (8, 24)), p = 0.10). The same was true when functions for final discrimination performance over the last 8 days (DP, not shown in figure) where analysed: Again, no significant differences were seen between lesioned and control groups. Neither the two nonlinear regression analyses (Left vs. control: Beta = 0.07, t(430) = 1.90, p = 0.06; Right vs. control: Beta = 0.06, t(400) = 1.71, p = 0.06) nor the Mann-Whitney U-tests (Left (13 (6, 20)) vs. control (10 (4, 16)), p = 0.07; Right (11(3, 17)) vs. control (16 (5, 22)), p = 0.08) showed any significant differences between lesion and control groups.

By contrast, unilateral AC lesions showed effects on the learning performance of those animals that were trained to discriminate fast periodicities of 160 vs. 320 Hz (Fig. 2 lower panels, red curves): The slope of the CR+-curve, a measure of LS, was less steep in the left lesioned group compared to the control group. This difference was significant (Nonlinear regression analysis: Beta  = −0.24, t(489) =  −6.51, p<0.001); Mann-Whitney U-test across the first seven training sessions: Left ((8 (4, 16)) vs. control (14 (9, 19)), p<0.001). Furthermore, the final DP reached during the last 8 days was lower in the lesioned compared to the control group, pointing to a discrimination learning impairment introduced by the left AC lesion (Nonlinear regression analysis: Left vs. control: Beta = −0.27, t(434) =  −7.19, p<0.001) Mann-Whitney U-test across the first seven sessions: Left (2 (0, 6)) vs. control (5 (2, 10)), p = 0.002).

By contrast, in the right AC lesioned group the final DP was higher than in the control group, suggesting an improvement of final discrimination learning performance after right AC lesion in this task (Nonlinear regression analysis: Right vs. control: Beta = 0.15, t(432) = 3.72, p<0.001; Mann-Whitney U-test across the first seven sessions: Right (7.5(2, 15.5)) vs. control (5(1,8)), p = 0.01).

**Figure 3:**
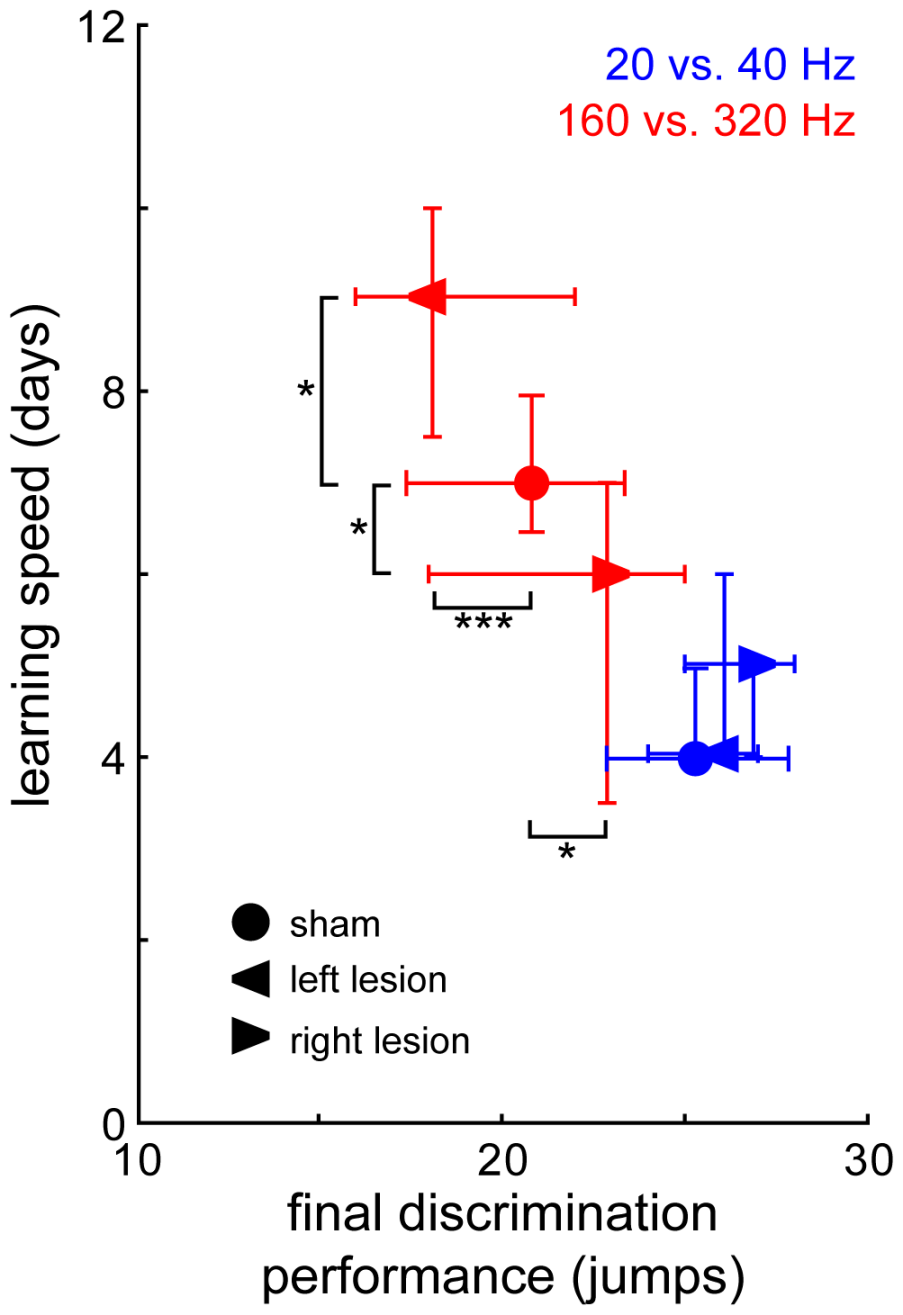
Comparison of two measures of learning performance, learning speed (LS) and final discrimination performance (final DP). LS is identified as the first day with significant performance, i.e. with a significant difference of CS+ and CS– induced hurdle jumps (fourfold table χ2-test, note that a smaller value reflects faster learning), final DP is a measure of how well the animals finally learn to discriminate the stimuli and is defined as the median of the differences between CR+ and CR- across the last 8 training sessions of each individual animal (cf. Methods). Given are median values and quartiles for animals that were trained to discriminate periodicities of 20 Hz vs. 40 Hz (blue data points) or 160 Hz vs. 320 Hz (red data points). Between-group comparisons for LS and final DP were carried out using Bonferroni-corrected Mann-Whitney U-statistics, p-level:.*p<0.05, ***p<0.001.

Correlations of our measures for LS and final DP (cf. Methods) further supported these hemispheric differences in the effects of unilateral AC lesion on discrimination learning of fast AM tones. This is depicted in Figure 3 where both parameters (LS and final DP) are plotted against each other for all groups. In addition, separate Mann-Whitney U-tests were performed to compare each single parameter of the lesion groups with their control groups. No significant effect of unilateral AC lesion on discrimination learning of slow periodicities was found neither in LS nor in final DP (blue data points). In contrast, left AC lesion led to a significant impairment of LS in comparison to controls when fast periodicities had to be discriminated (red data points; Mann-Whitney U-test, p = 0.035) while right AC lesion led to a significant improvement of LS in comparison to controls (Mann-Whitney U-test, p = 0.016). In accordance with these results, left AC lesion also significantly impaired final DP in this task (Mann-Whitney U-test, p<0.001) and right AC lesion also significantly improved DP (Mann-Whitney U-test, p = 0.036). In other words, left AC lesion impaired not only LS but also final DP in the learning of fast modulated AM stimuli discrimination while on the contrary lesion of the right AC improved both parameters. Neither AC lesion was affecting these learning parameters in the slow modulated AM discrimination task.

**Figure 4:**
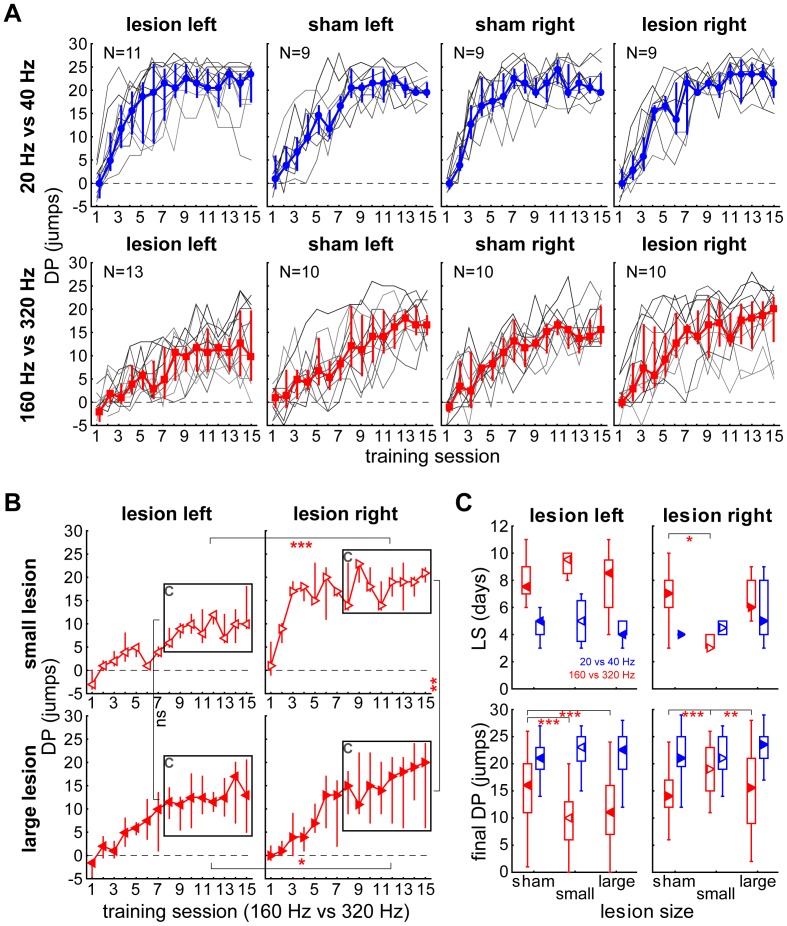
Effect of lesion size on discrimination performance (DP), final DP and learning speed (LS). A DP of all 83 animals (lines in shades of grey) over all training sessions in the 8 different groups with animals trained to discriminate periodicities of 20 Hz vs. 40 Hz (medians of data and quartiles in blue) and 160 Hz vs. 320 Hz (medians and quartiles in red). **B** Comparison of the DP of lesioned animals in 160 Hz vs. 320 Hz paradigm divided by lesion size (small lesion: open symbols; large lesions: solid symbols). Given are the DP median values and quartiles (whiskers), the boxes indicate the values used for the calculation of the final DP used in C. Between-group comparisons were carried out using Bonferroni-corrected Mann-Whitney U tests, p-level: *p<0.05, **p<0.01, ***p<0.001. **C** Comparison of LS and final DP dependent on lesion side and size. Given are median values (arrows), quartiles (boxes) and the full range of values (whiskers) of LS (upper panels) and final DP (lower panels) for animals that were trained to discriminate periodicities of 20 Hz vs. 40 Hz (blue data) or 160 Hz vs. 320 Hz (red data) and received either a sham lesion or a left or right sided lesion of different extension. Between-group comparisons of lesion size effects were carried out using Kruskal-Wallis ANOVA, p-level: *p<0.05, **p<0.01, ***p<0.001.

These differences are also obvious when looking on the daily discrimination performance over all training sessions for all individual 83 animals (Figure 4A, grey lines) in all 8 behavioural groups (control groups of left and right sham surgery show separately). The majority of the animals behaved similar to each other within their groups (low interquartile range deviation from the median values; blue and red lines and symbols in Figure 4A) with some exceptions especially in the lesion groups of the fast discrimination paradigm (Figure 4A lower left and lower right panel).

To explain these increased variances, we investigated whether lesion size had any effect on the observed hemispheric learning differences (Figure 4 B and C). For this purpose, we divided the lesioned groups (cf. Figure 2) into two sub-groups each, comprising the animals with the smallest (<7 mm^3^) or largest (>7 mm^3^) lesions, respectively. This threshold was chosen from a generalized k-means cluster analysis of lesion size that resulted in a cluster of small lesion sizes (centroid mean 4.4 mm^3^, upper cluster limit 6.89 mm^3^) and a large lesion cluster (centroid mean 10.8 mm^3^, lower cluster limit 7.77 mm^3^). The size of the lesions of left and right hemispheres were not significantly different between the corresponding slow and fast AM discrimination paradigms (Mann-Whitney U-tests, always p>0.05). To compare the lesion size dependence of final DP in the two groups with the largest variance in the 160 Hz vs 320 Hz paradigm we performed Mann-Whitney U-tests on the DP of the last 8 days ( =  final DP) and corrected them for multiple comparisons (Figure 4B). In the animals with lesions on the left side we found no difference in final DP for small and large lesions. Animals with lesions on the right side did show differences in final DP in this paradigm depending on the size of the lesion: Small right side lesions led to better learning than large right side lesions (Mann-Whitney U-test, p = 0.008) and to a larger final DP than small left side lesions (Mann-Whitney U-test, p<0.001). This was also true for large right side lesions that enabled the animals to learn better than after large left side lesions (Mann-Whitney U-test, p = 0.018). Comparing all groups depending on their lesion size in LS and final DP in Figure 4C a similar picture arose. As described above (cf. Figure 3) no effect of unilateral AC lesion of any size was seen for the slow AM training paradigm (blue data in Fig. 4C; Kruskal-Wallis ANOVA, criterion: α = 0.05). By contrast, for the discrimination learning of fast AM periodicities (red data), there was a significant size effect. Yet unexpectedly, improvements of LS and final DP after right AC lesions were stronger for small lesion sizes as seen in Fig. 4C. In fact, for LS only small lesion sizes in the right hemisphere resulted in significant differences to sham controls (Kruskal-Wallis ANOVA, H (2, N = 32) = 6.79, p = 0.031). The effect was abolished with large lesions of right AC, i.e., no significant difference to the control group could be detected for lesion sizes larger than 7 mm^3^. For left AC lesions there was no significant impairment of LS compared to controls in any of the subgroups. As this is different from the impairment of the total lesion group, this is a statistical effect due to splitting of the group.

For final DP, significant effects were seen for both left and right AC lesions: For left AC lesions, Kruskal-Wallis ANOVA demonstrated significant impairment of final DP for both lesion sizes (H (2, N = 184) = 22.89, p<0.001; with small and large lesion vs. control: p<0.001 each), with no significant difference between lesion sizes (p = 0.65). But for right AC lesions a significant effect of lesion size on final DP was found (H (2, N = 176) = 16.55, p<0.001). Significant improvements of final DP were seen only for small (p<0.001) but not for large lesion sizes (p = 0.99). In summary, small lesions of right AC improved discrimination learning speed and final discrimination performance, whereas large lesions abolished this effect. By contrast left side lesions impaired fast AM discrimination irrespective of the lesion size.

**Figure 5:**
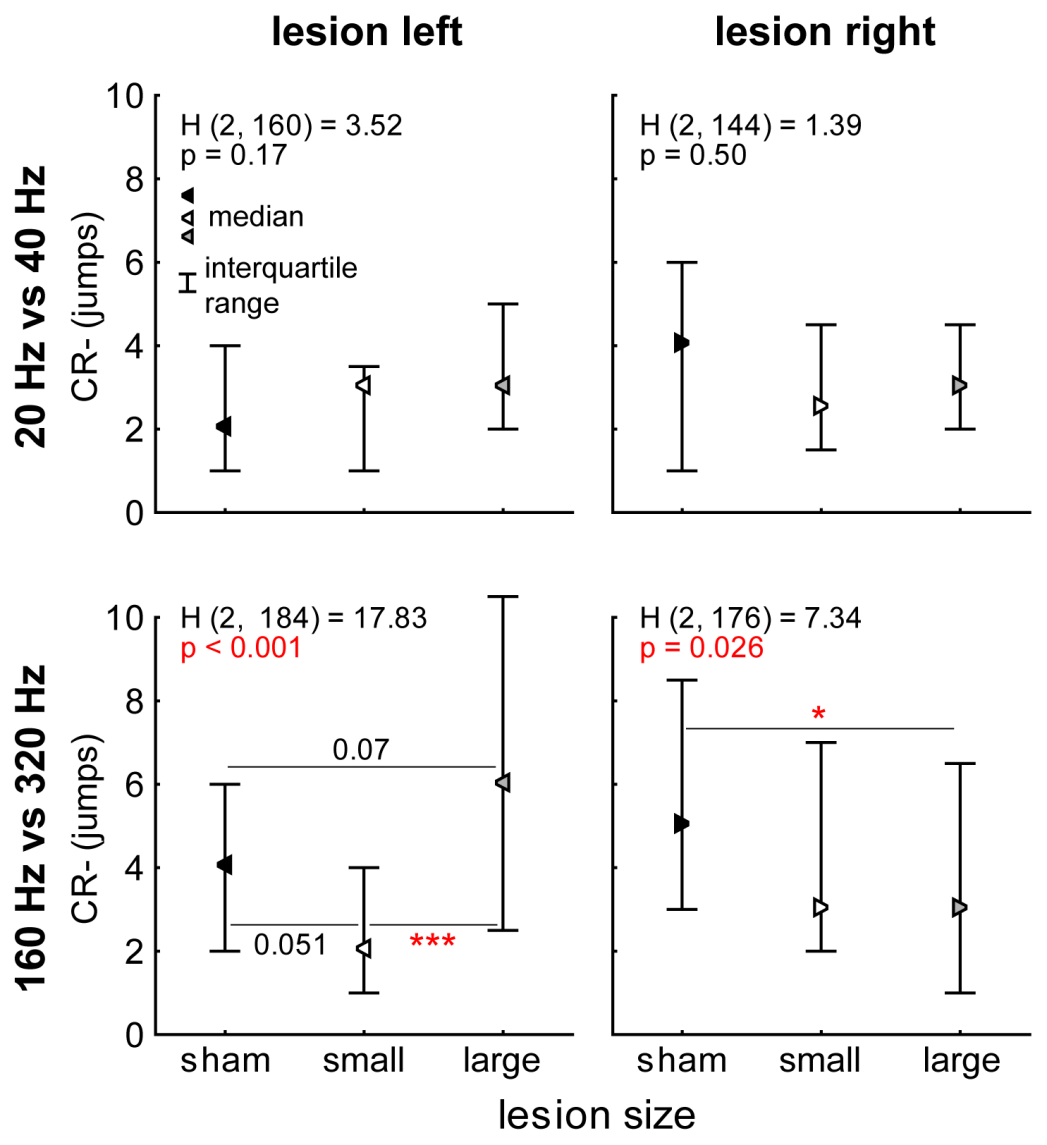
Effects of lesion size on CR-. Given are the median values of wrong jumps over the hurdle (false responses) from day 8 to 15 (stable DP phase) for sham control animals and animals with small and large lesions of the left and right AC during slow (upper panels) and fast AM discrimination learning (lower panels). Whiskers indicate the interquartile range. Between-group comparisons of lesion size effects were performed using Kruskal-Wallis ANOVA and post-hoc multiple comparisons of mean ranks were used to investigate differences between two groups; p-levels: *p<0.05, ***p<0.001.

In general, fast AM stimuli as used in this study potentially contain two different perceptual cues that may be used for discrimination, namely periodicity pitch and timbre where periodicity pitch is believed to be a comparably strong cue which is predominantly coded in the left AC while timbre is generally thought to be a weak cue that is predominantly coded in the right AC [17], [18]. To investigate a possible interference of these two cues in the animals’ decision making we analysed the false response rates (CR-) in more detail. Figure 5 depicts the results of this analysis. The upper two panels show the CR- from day 8 to 15 (in the stable phase of DP) for the slow AM modulations in left and right lesioned animals. The Kruskal-Wallis ANOVAs indicate no significant differences between sham control and small and large lesioned animals (left: H(2,160) = 3.52, n.s.; right: H(2,144) = 1.39, n.s.). This is also true if both lesion groups are taken together and tested with a Mann-Whitney U-test vs. sham controls (left: p = 0.15; right: p = 0.24). On the other hand in fast AM stimuli we found strong effects of lesion size on the CR-; if the animals’ left AC was lesioned (periodicity pitch coding impaired) the significant difference as demonstrated by Kruskal-Wallis ANOVA (H(2,184) = 17.83, p<0.001) allowed multiple comparisons of mean ranks which in turn showed a tendency for small left lesions (p = 0.051) allowing the animals to make less mistakes compared to sham controls. Large left lesions tended to lead to more mistakes than in control animals (p = 0.07). The difference between both lesion groups was highly significant (p<0.001). When both lesion groups were taken together, the effect of the lesion vanished (Mann-Whitney U-test, p = 0.70). Lesion of the right AC (timbre coding impaired) also resulted in a significant difference in the Kruskal-Wallis ANOVA (H(2,176) = 7.34, p = 0.026) indicating a dependency of CR- on lesion size, which was a significant decrease in wrong responses for large lesions vs. sham controls (multiple comparisons of mean ranks, p = 0.048). Grouping both lesion groups into one also resulted in a significant better CR- in the lesioned animals compared to controls (Mann-Whitney U-test, p = 0.007). In other words, animals with lesion of the right AC did not perceive an ambiguous stimulus because only periodicity pitch as a strong cue could be coded and by that made fewer mistakes than healthy controls. Impairing the left AC enabled the animals only to rely on (weak) timbre cues for discrimination which did not lead to significant improvement in behaviour.

## Discussion

In the present report we have demonstrated that the ability to learn discrimination of fast periodicities of AM tones is predominantly represented in the left AC, whereas unilateral AC ablation on either side has no effect on the discrimination of slow periodicities. This latter result is in line with earlier studies on periodicity coding in the Mongolian gerbil which showed that two different codes for periodicity representation of fast and slow AM are realized in AI [10] that are also reflected in different learning behaviour [11]. Out of these only the discrimination learning of fast periodicities is dependent on cortical processing whereas for the discrimination of slow periodicities sub-cortical processing seems to be sufficient [7].

A lateralization of brain functions to a dominant hemisphere as described in this report for discrimination learning of fast periodicities is not a unique result, in a number of studies, similar lateralization phenomena have been described. For example, Wetzel and co-workers [6] have described that right but not left AC lesion impairs discrimination of rising vs. falling frequency-modulated tones (FM) in Mongolian gerbils, a finding that could be replicated in rats [19]. Left-lateralized AC fields specialized for the perception of species-specific communication calls have been described in mice [20] and macaques [21], reminding of the typically left-lateralized cortical fields for speech processing in humans [17], [22]. On the other hand, these left lateralized specializations may not be directed at the category of species-specific vocalizations per se but at temporal features which are characteristic of such vocalizations and various other sounds. E.g. it has been found that left AC alone can discriminate rising from falling FM if the continuous FM sweeps are fragmented into a series of shorter sweeps, i.e. by introducing temporal sequence cues into the stimuli [13].

Most studies about lateralization phenomena in the AC of animals and humans point to a different auditory specialization of the two hemispheres, where the left hemisphere seems to be more capable of analysing temporal resolution and sequence aspects of sounds whereas the right hemisphere seems to have advantages in analysing spectral contours of sounds [3], [13], [18], [23]. This hemispheric specialization of AC for the analysis of different aspects of sound may be realized by the implementation of different principles of cortical microarchitecture realizing different patterns of neuronal interconnectivity that are ideal for different types of neuronal processing [24], [25], [26]. This divergent neuronal microarchitecture of the two hemispheres may be an obligatory consequence of the need to simultaneously analyse qualitatively different features of the same sounds. This is a view that is different from a simple hemispheric dominance concept where both ACs are capable of performing the same analysis but one better than the other. Our result of a left-hemisphere advantage for the processing of fast AM tone periodicities fits into this scheme of AC hemispheric specialization.

Schneider et al. [27] reported for humans that the decoding of pitch according to the fundamental frequency of harmonic complex sounds is lateralized to the left hemisphere. Although the AM tones with fast periodicities used here were not harmonic and we do not know if gerbils do perceive a periodicity pitch, one may still argue that gerbils analyse modulation frequencies of fast AM in the temporal domain. In the light of our earlier findings of a rate-place periodicity map in gerbil left AC [9], [10] and the fact that the AC is crucial for the discrimination of fast AM tone periodicities in gerbils [7], one may conclude that the fundamental is analysed by temporal, subcortical mechanisms and the result of this analysis is then topographically mapped onto AI via a non-temporal rate-place code, that in turn is used (and required) for behavioural readout.

The fact, that there was no hemispheric specialization obvious for the discrimination of 20 vs. 40 Hz may indicate that this task is not demanding enough to require involvement of cortical processing. Indeed, as demonstrated earlier, this task can be solved by the animals without AC, namely after bilateral AC ablation [7].

More intriguing than the mere fact that processing of fast AM periodicities is lateralized to the left AC is the finding that lesion of the right AC led to an improvement in this type of auditory learning task.

There are only a few reports of improvements of a brain function after brain lesion, the best-known of which is the so-called Sprague-effect [28]. It describes the effect that the impairment of visual orienting responses that follow a hemianopia due to the unilateral ablation of the visual cortex of the contralateral hemisphere can be completely restored by additional removal of the tectum contralateral to the cortical lesion. This Sprague-effect which initially was described for cats has in the meantime been extended to humans [29] and even to the auditory orientation system [30].

In contrast to the Sprague-effect, where an impaired function after cortical lesion is restored by an additional (tectal) lesion, we have found a phenomenon where a cortical lesion led to an improvement of a function compared to the unlesioned control group performance. A similar finding was reported for the Stroop-interference [31] where the naming speed of words written in colour is reduced if a word names a deviant colour: Pujol and co-workers [32] reported that patients with right frontal demyelination showed impaired Stroop responses whereas lesions in the left posterior parietal cortex improved performance in the Stroop-test above normal performance.

Such results suggest some kind of competitive interaction between brain regions. This may result in improved performance of a certain neuronal function if the competitive influence on the region where this function is optimally implemented is abolished by lesion of the competing region. This interaction of regions may be realized by direct projections between such regions, or by projections of both regions onto a common brain region that e. g. controls the behaviour observed.

In our experiments on discrimination learning of fast AM tones, a simple division of labour between the two hemispheres focussing on spectral or temporal processing, like reported by Wetzel et al. [13] for FM, would not be sufficient to explain the effect of right lesion-induced better than normal discrimination performance. The two auditory cortices normally interact through the corpus callosum but it would not make much biological sense that a spectral analysis of sounds by right auditory cortex generally impairs the simultaneous temporal analysis of the sound in the left auditory cortex. However, a competition is conceivable, e.g. if sounds to be discriminated in a learning experiment contain several different cues that all potentially lead to a correct behavioural classification.

During discrimination learning in a shuttle box, animals have to learn through trial and error with reinforcement feedback that one stimulus means “jump” and the other “no jump”. The basis of this associative learning is the ability to discriminate the two stimuli. If the stimuli have only one distinguishing feature for processing, e.g. different pitch of pure tones, there is no ambiguity of what to discriminate. But in the case of 160 vs. 320 Hz AM, both with a carrier of 2 kHz, there are at least two different perceptual cues to distinguish these stimuli, periodicity pitch and timbre. Analysis of periodicity pitch has been attributed to a left AC region, at least in the human brain [27] while analysis of timbre, mainly due to spectral properties of complex sounds, and spectral pitch has been attributed to right AC [33], [34], although none of these functions seems to be completely lateralized to either side [35]. The fact that left AC lesions do not lead to a complete failure but rather to a quantitative impairment of the AM discrimination learning in comparison to intact brains may suggest that timbre difference is a cue that can also lead to discrimination (cf. Figure 5). But this cue seems to be clearly inferior with respect to performance to the cue used by the left hemisphere when the right AC is lesioned, i.e. presumably the periodicity pitch. This can be concluded from the result that discrimination learning with left AC alone is better than with right AC alone. A possible inference from these considerations is that in the normal case where intermediate performance is observed, there might be a competitive interaction between hemispheric cue processing through the corpus callosum. The hypothesis we want to propose here for the normal case is that throughout the learning the ambiguity between the two cues which may not be fully resolved increases the mistake rate. Several possibilities arise how such interactions may influence the rate of mistake during the progression of discrimination learning, e.g. attention switching between the cues or alternatives of forming associations between the cues and the correct and incorrect behaviours. Even that the pitch cue and the timbre cue correlate for the identification of a given AM needs to be found out by the animal through trial and error. Therefore correctly attributing e.g. a GO-meaning to a given AM on the basis of pitch may not lead automatically to the acceptance of the corresponding timbre for this GO-meaning. Consequently, the probability of mistakes might be larger if the two hemispheres interact during the associative learning than for the use of one cue (the pitch cue) alone. Our data of the CR- of the lesioned animals (Figure 5) seem to support this view. To further test this hypothesis, a learning paradigm could be designed where the animals are trained to discriminate stimuli where the pitch and the timbre cue correlate for the identification of CR+ and CR-, as it was done here. After the animals’ behaviour shows stable discrimination performance, their behaviour might be analysed in a test session, where new stimuli are presented with ambiguous combinations of the pitch and the timbre cue.

Finally, our result that small lesions of the right AC led to improvement of AM discrimination learning whereas large AC lesions abolished this effect, suggests that in addition to the discussed cue-specific interaction between hemispheres also other interactions between AC fields and with other brain structures may play an important role. Possibly, small lesions centred to AI did not include all AC fields, and large lesions did include cortical fields surrounding AI. In the first case, only a partially ablated right AC centred to AI would lead to a relief of competition with left AC function because the timbre cue no longer interacts with the pitch cue. On the other hand, due to a complete right AC lesion, no interaction between left and right AC and no output from right AC to other brain structures is possible. This may weaken even the advantage of the left AC for behavioural task performance to the extent that the improvement by the small right AC lesion is abolished. To test these hypotheses, the ablation experiments as performed here might be combined with the known functional labelling of gerbil AC fields, e.g. using the 2-deoxyglucose-method [14], to analyse in detail which AC fields are responsible for the improvement effect.

From a mere perspective of imbalances of hemispheric interactions it seems that supranormal performance in certain tasks may only come at the cost of impairment in other tasks. The most extreme examples of this probably represent certain forms of autism (savantism). In our model, the Mongolian gerbil, the improvement in AM discrimination learning after right AC lesions comes at the cost of a loss of discrimination ability of another class of stimuli, namely FM [6], [13].
